# A Structural Model Approach to Ethnic Disparities in Dementia and Its Assessment

**DOI:** 10.1093/geroni/igaa057.3122

**Published:** 2020-12-16

**Authors:** Donald Royall

**Affiliations:** UTHSCSA, San Antonio, Texas, United States

## Abstract

Ethnicity complicates the assessment of dementia and its biomarkers in the Texas Alzheimer’s Research and Care Consortium (TARCC). Its effect can be mitigated by the construction of latent variables in a structural equation model (SEM) framework. We have developed a dementia-specific phenotype, i.e. “δ” (for “dementia) by that approach. δ provides a continuously distributed dementia severity measure that may be resistant to ethnicity effects. We propose to test the impact of Mexican-American (MA) ethnicity in TARCC data [N = 3502; MA = 1313; Non-Hispanic Whites (NHW) = 2189]. Significant structural associations between observed cognitive performance, δ and δ’s serum protein biomarkers will be tested for ethnicity effects by CHI SQ differences across ethnicity stratified models. Observed clinical variables can be impacted by demographic effects. Those can lead to presumed disparities in clinical outcomes or biomarkers. Latent variables have potential to mitigate demographic effects, refute some perceived disparities and confirm others.

